# Antibacterial and anti-inflammatory efficacy of N-acetyl cysteine in endodontic treatment: a scoping review

**DOI:** 10.1186/s12903-022-02433-6

**Published:** 2022-09-12

**Authors:** Saleem Abdulrab, Nawras Mostafa, Sadeq Ali Al-Maweri, Hisham Abada, Esam Halboub, Hatem A. Alhadainy

**Affiliations:** 1 Um Salal Health Centre, Primary Health Care Corporation, Doha, Qatar; 2 Department of Restorative Dentistry, Faculty of Dentistry, University of Western Cape, Cape Town, South Africa; 3 Department of Endodontics, Faculty of Oral and Dental Medicine, Kafrelsheikh University, Kafr El-Sheikh, Egypt; 4 Department of Maxillofacial Surgery and Diagnostic Sciences, College of Dentistry, Jazan University, Jazan, Kingdom of Saudi Arabia; 5 Department of Oral Medicine, Oral Pathology and Oral Radiology, Faculty of Dentistry, Sana’a University, Sanaa, Yemen; 6 Department of Endodontics, College of Dentistry, University of Tanta, Tanta, Egypt

**Keywords:** N-acetyl cysteine, Endodontic treatment, Root canal disinfection, Scoping review

## Abstract

**Background:**

This scoping review systematically summarized the available evidence about the efficacy of N-acetyl cysteine (NAC) as an intracanal antibacterial and/or anti-inflammatory.

**Methods:**

PubMed, Scopus, Web of Science, and Google scholar search engines/databases were searched up to February 2022 to retrieve relevant studies. The studies were evaluated for eligibility criteria, and identifying relevant studies.

**Results:**

Out of 193 studies, 15 fulfilled the inclusion criteria and were processed for data extraction. Thirteen in vitro studies assessed antibacterial/antibiofilm efficacy of NAC, and reported good and promising efficacy: NAC was found as efficacious as the comparators (chlorhexidine, sodium hypochlorite, calcium hydroxide), or even showed higher efficacy. Regarding the anti-inflammatory efficacy of NAC, one in vitro study found it equivalent to, while one clinical trial revealed it more efficacious than calcium hydroxide.

**Conclusions:**

There is accumulating evidence on the anti-microbial and anti-inflammatory efficacy of NAC in context of endodontics. However, further clinical trials with robust methodology and objective and reliable clinical, biological and microbial outcomes are warranted to translate its use for clinical practice on humans.

## Background

Pulpal and periapical diseases are caused mainly by the presence of microorganisms, mainly bacteria, and their by-products [1, 2]. Once the root canal system is infected, bacteria will be present as either free-floating (planktonic) single cells or biofilms which are sessile multicellular microbial communities adherent to each other and embedded in a 3D matrix of self-produced extracellular polymeric substances (EPS) [3]. The success of endodontic treatment depends on the elimination of the microorganisms from the root canal system or, at least, their reduction below the threshold level that is compatible with the healing of periapical tissues and prevention of reinfection [4, 5].

*Enterococcus faecalis* (*E. faecalis*) is the most commonly isolated microorganism from infected root canals. *E. faecalis* dominates in up to 90% of the secondary and persistent infections, although its prevalence is surprisingly less by nine times in primary infections [6]. These figures explicitly indicate the role of *E. faecalis* in the failure of endodontic treatment. The virulence of *E. faecalis* is claimed to be due to its resistance to intracanal medication [7, 8], and ability to survive in a poor environment without support of other bacteria [9, 10], along with its ability to produce biofilms and hence it becomes more resistant to antibodies, phagocytosis, and antibacterial agents [10]. *Streptococcus mutans* is another species that could be present in endodontic infections that further complicates the situation as it interacts with other microbial communities, enhances biofilm formation [11], and increases resistance to intracanal medication [12].

The diversity of the microbial community of root canal infections and its ability to form biofilm make it necessary to use irrigation materials (during cleaning procedure) and intracanal medications (between visits); axiomatically these materials should have antimicrobial and/or anti-inflammatory properties [4]. In the context of endodontic treatment, up to 35% or more of the root canal surfaces remain un-instrumented even with the most efficient instrumentation techniques; this simply means that the microbial biofilms are not disrupted in these areas. Other irregularities like lateral and accessory canals, fins, cul-de-sacs, and isthmus might also remain un-instrumented, and hence the formed microbial biofilms there remain undisrupted [13]. Fortunately, the formed microbial biofilms in these inaccessible-for-instrumentation areas can be removed or, at least, reduced by the irrigation fluid [4], and this is highly recommended to enhance the success rate of root canal treatment [14]. The ideal irrigation and/or intracanal medication should have numerous desirable properties, such as being antimicrobial, biocompatible, in addition to having favorable physical properties.

There is growing evidence that the biofilms of oral bacteria are more resistant to antimicrobial agents such as chlorhexidine (CHX), amine fluoride, vancomycin, ampicillin, doxycycline, amoxicillin, metronidazole, and linezolid compared with planktonic cells [15, 16]. Growing evidence exists that bacteria in biofilms, including *E. faecalis,* couldn’t be completely eradicated and/or killed with 2% CHX solution and 1% and 3% sodium hypochlorite (NaOCl) [17]. So, the ideal irrigating solutions and intracanal medications must be able to dissociate the biofilm building blocks (the EPS), in addition to having antimicrobial activity to guarantee the complete elimination of the biofilm.

In the context of endodontic treatment, NaOCl is considered an effective antibacterial agent, good lubricant, and great organic solvent. Hence, it is the most commonly used irrigating solution [18]. According to Clegg et al. [17], 6% NaOCl irrigant is capable of rendering bacteria nonviable and eliminating the biofilm. However, NaOCl in high concentration is extremely irritating to the periapical tissues [19]; causes dentin deproteination, and collagen breakdown; and decreases the flexural strength of dentin [20]. CHX at a 2% concentration is also used as an irrigant [21]. It possesses an antibacterial effect against Gram-negative and Gram-positive bacteria, with therapeutic qualities providing long-term benefits.

N-acetyl cysteine (NAC) is a thiol-containing drug with antioxidant and mucolytic properties rendering it a good candidate for medical treatment of acetaminophen overdose and chronic bronchitis, respectively [22, 23]. Although it is a non-antibiotic chemical compound, it has antibacterial capabilities. To cite examples, NAC inhibits biofilm formation by gram-positive and gram-negative bacteria [24, 25]; reduces extracellular polysaccharide formation effectively; disrupts established biofilms; and decreases bacterial adhesion to surfaces [26, 27]. The antioxidant property of NAC is ascribed to the ease by which it is absorbed into the cells where it immediately neutralizes reactive oxygen species [28]. Another property that makes NAC magical is that it exerts anti-inflammatory activity by inhibiting the expression and release of a variety of pro-inflammatory cytokines that have been associated with inflammatory tissue [29].

In the context of endodontics, NAC has been proven efficient in killing both planktonic and biofilm forms of *E. faecalis* at pH 11 [30]. Its biofilm-disrupting property comes from its interfering effect on the synthesis of EPS. A study has shown that NAC suppresses *E. faecalis* biofilm development and eliminates it [30]. Another study showed that the antibacterial effect of NAC is higher than that of NaOCl and CHX. More specifically, 200 mg/ml solution of NAC was found to be more efficient than 5.25% NaOCl and 2% CHX in killing *E. faecalis* and *S. mutant* bacteria [31]. More recent studies reported that the application of NAC as intracanal medication considerably elevated resolving E1 and D2 levels which are potent endogenous anti-inflammatory mediators [32], and reduced TNF-α which is a potent inflammatory cytokine [33].

Given the scarcity of information on the effect of NAC as an irrigant and/or intracanal medication, and the lack of systematic or scoping review on the same, this study aimed at summarizing systematically the available evidence about the efficacy of NAC as an intracanal antibacterial and/or anti-inflammatory.

## Materials and methods

The guidelines of the Preferred Reporting Items for Systematic reviews and Meta-Analyses extension for Scoping Reviews (PRISMA-ScR) were followed to answer the study question/objective: The antibacterial and/or anti-inflammatory efficacy of NAC as root canal irrigating solution and/or medication.

### Search strategy

PubMed, Scopus, Web of Science, and Google scholar search engines/databases were searched since the date of inception up to February 2022. The following keywords were used: (“N acetylcysteine” OR “N-acetylcysteine” OR NAC) AND (“endodontic treatment” OR (“root canal pathogens” OR “root canal bacteria” OR “root canal microorganisms” OR “endodontic bacteria” OR “endodontic microorganisms” OR “endodontic infection” OR endodont* OR “root canal disinfection” OR “root canal treatment” OR “root canal infection” OR “intracanal disinfection” OR “root canal medicaments”)).

### Eligibility criteria

This scoping review involved all studies in the English language, including clinical and in-vitro studies. Only studies where antibacterial and/or anti-inflammatory effects of NAC were compared to other endodontic irrigants and/or medicaments were included. Studies without control groups, case reports, case series, and review studies were excluded.

### Identifying relevant studies

An electronic de-duplication method was implemented using EndNote X9 citation management system. The titles and abstracts of the remaining records were screened independently by two authors (NM and SAA). Disagreements, if any, were resolved via consultation with a senior author (SA). The full texts of the remaining potentially relevant studies were comprehensively read for further confirmation of relevancy to the study question. The relevant studies that fulfilled the eligibility criteria were processed for data extraction.

### Data charting process and data items

Two authors (NM and SA) independently extracted the necessary information. The following data were extracted from each study: authors and year of the article, country, study design, sample (number and type of teeth), application method, targeted bacteria, assessment methods, and the reported results.

## Results

### Selection of sources of evidence

**Fig. 1:**
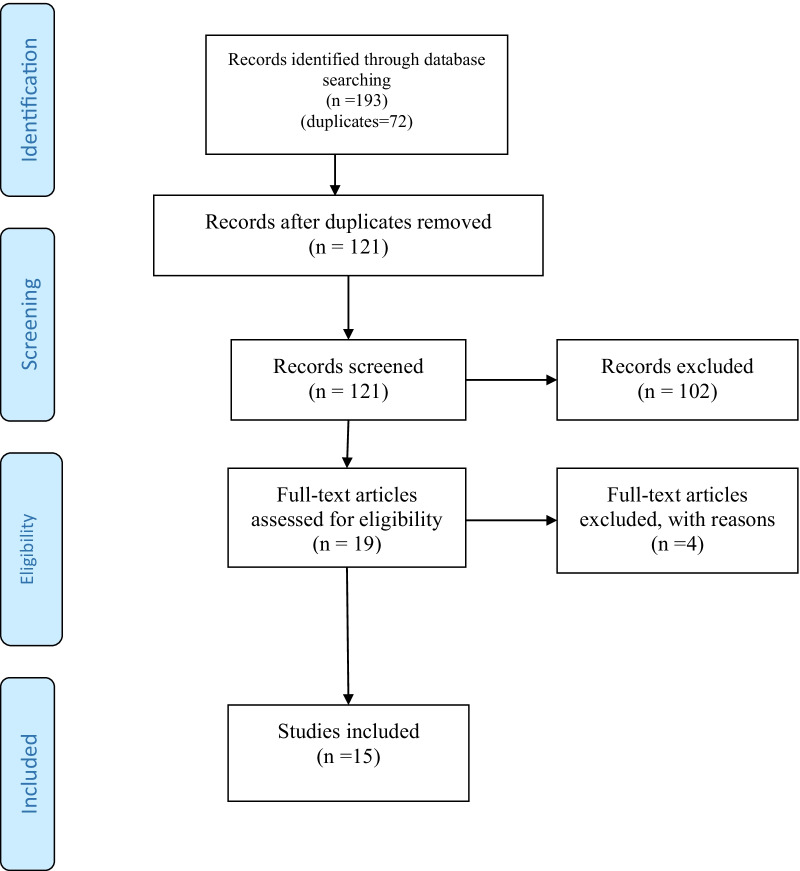
Flow diagram of the screening and selection process adapted from the Preferred Reporting Items for Systematic Reviews and Meta-Analyses (PRISMA) statement

A total of 193 articles were retrieved from online searches (PubMed = 25, Scopus = 28, Web of Science = 40, Google scholar = 100 [top 100 relevant studies]). The electronic de-duplication removal of duplicates resulted in excluding 72 articles. After an independent screening of the titles and abstracts of the remaining 121 records, 102 were excluded. After an independent and comprehensive reading of the full-texts of the remaining 19 articles, four were excluded as being irrelevant to the study question. Ultimately, 15 studies fulfilled the inclusion criteria and were processed for data extraction. Figure 1 depicts the results of the search process.

### General characteristics of the included studies

Table 1 presents comprehensive details on the characteristics of the included studies. In brief, a total of 15 studies (555 teeth) were included in the present review [14, 30–43]. Of these 12 were in-vitro studies [14, 30, 31, 33–41] and one was a randomized clinical trial [32]. Three of these studies were conducted in India [34, 38, 39], two in Korea [14, 37], two in Turkey [33, 35], two in Brazil [32, 40], two in Iran [41, 43], and one each in Egypt [31], Singapore [30], Indonesia [36] and Spain [42]. Ten studies [14, 30, 31, 34, 35, 37–41, 43] included sound extracted single-rooted teeth (central incisors or premolars) while three studies [14, 36, 42] took the bacterial sample directly from patients with non-vital teeth. The sample size differed greatly among the included studies, ranging from 16 to 120 teeth [14, 30, 31, 34, 35, 37–39, 41].

**Table 1:** Characteristics of the Included Studies

Author	Study design	Intervention and type of application	Control group	Type and number of teeth	Outcome measure	Outcome measures (methods)	Exposure Volume	Exposure time	Main outcomes
Quah et al. 2012 [30] (Singapore)	In-vitro	NAC 200 mg/mL (irrigation)	CH	30 Human premolars	Microbial *E. faecalis*	CFU Dead cells (CLSM)	NA	7 days	NAC (12.5 mg/mL) was significantly more efficacious in killing *E. faecalis*, regardless of dentin powder pre-incubation compared to CH where pre-incubation with dentin powder abolished the antibacterial effects of CH (*P* < .05)
Darrag 2013 [31] (Egypt)	In-vitro	NAC 200 mg/mL (irrigation)	G1:sterile distilled water G2: 5.25% NaOCl G3: 2% CHX	40 Single rooted teeth	Microbial *E. faecalis* *S. mutants*	CFU CLSM	5 ml	5 min	Both planktonic bacteria were more sensitive to NAC solution recording significantly the lowest mean CFU values compared to controls. The results also showed significantly more proportion of dead cells in dual-species biofilm when NAC irrigation solution was used
Ulusoy et al. 2015 [35] (Turkey	In-vitro	NAC 200 mg/mL (medicament)	G1:CH 0.1% G2: CHX 2% G3: NaOCl 5.25% G4: Taurolidine 2% (medicament)	70 Human premolars	Microbial *E. faecalis*	CFU SEM MTT assay	NA	7 days	All groups showed a good efficacy. However, CH was the strongest bactericidal agent at all test dilutions, regardless of the presence of dentin powder. The antibacterial effect of NAC and taurolidine was significantly lower than that of CH at all test dilutions
Moon et al. 2016 [14] (Republic of Korea)	In-vitro	NAC 200 mg/mL (medicament)	G1: sterile saline G2: Saturated CH G3: 2% CHX	Hydroxyapatite (HA) disks	Microbial *A. naeslundii* *L. salivarius* *S. mutants* *E. faecalis* (Multispecies biofilm	ATP-bioluminescence SEM	NA	10 min 24 h	The viability of mature biofilms was reduced by > 99% compared with CH or 2% CHX solution. Moreover, NAC was more efficacious in disrupting biofilm activity compared to saturated CH or 2% CHX
Planiswary et al. 2016 [34] (India)	In-vitro	G1: NAC 200 mg/mL G2: Combination of NAC and 2% CHX (irrigation)	G1: Saline G2: 2% CHX	16 Single rooted teeth	Microbial *E. faecalis*	Agar diffusion test CFU	NA	7 days	NAC and 2% CHX showed equivalent efficacy. Maximum inhibition was shown by a combination group of NAC and 2% CHX suggesting a synergistic action which is e highly significant (*P* < 0.001)
Choi et al. 2017 [37] (Republic of Korea)	In-vitro	G1: NAC 25 mg/mL G2: NAC 50 mg/mL G3: NAC 100 mg/m (medicament)	G1: Saline G2: 2% CHX G3: Saturated CH	27 Single-rooted premolars	Microbial *A. naeslundii* *L. salivarius* *S. mutants* *E. faecalis* (Multispecies biofilm	ATP-bioluminescence CFU SEM	NA	7 days	NAC showed better efficacy in biofilm cell removal and killing than saturated CH or 2% CHX solution. Furthermore, 100 mg/mL NAC disrupted the mature multispecies endodontic biofilms completely
Ridhalaksani et al. 2018 [36] (Indonesia)	In-vitro	G1: 200 mg/mL NAC pH 2.5 G2: 200 mg/mL NAC pH 11 (Irrigation)	G1: sterile saline G3: 2% CHX	NA	Microbial *E. faecalis*	CFU	NA	1 min	All tested groups showed good efficacy. However, NAC at pH 11 test group showed the greatest efficacy in t reducing the bacterial colonies, which was statistically significant when compared to the NAC pH 2.5 and 2% CHX groups
Bhasin et al. 2019 [38] (India)	In-vitro	NAC 200 mg/mL (Irrigation)	G1: 5.25% NaOCl G2: 2% CHX G3: Sterile distilled water	40 Permanent mandibular incisors	Microbial *E. faecalis* *S. mutans*	CFU CLSM	5 mL	5 min	NAC showed significantly better efficacy compared to sodium hypochlorite (NaOCl) group, 2% chlorhexidine and sterile distilled water groups
Singh et al. 2019 [39] (India)	In-vitro	NAC 200 mg/mL (Irrigation)	G1: Sterile distilled water G2: 2% CHX	60 Maxillary incisors	Microbial *E. faecalis* *S. mutans*	CFU	5 ml	5 min	NAC was significantly more efficacious than sterile distilled water and 2% CHX (*P* < 0.01)
Abu Hasna et al. 2020 [40] (Brazil)	In-vitro	G1: NAC G2: NAC + PDT (medicament)	G1: Saline G2: CH G3: PDT	80 Single rooted teeth	Microbial *E. faecalis*	CFU CLSM SEM	NA	NA	NAC is as effective as CH regardless of its combination with PDT
Alireza et al. 2021 [41] (Iran)	In-vitro	G1: NAC G2: CH + 5% NAC G2: 50% CH + 50% NAC (medicament)	G1: CH G2: DS 100 µg G3: CH + 5% DS G4: 50% CH + 50% DS	35 Single rooted mandibular premolars	Microbial *E. faecalis*	CFU	NA	7 days	All tested agents showed a good antibacterial activity compared to saline at both depths, i.e. 100 and 200 µm. NAC showed an antibacterial efficacy comparable to that of CH. However, both NAC and CH were inferior to DS
Karapinar et al. 2016 [33] (Turkey)	In vitro	NAC (medicament)	CH	Cell lines	LPS- induced Inflammatory mediators (TNF-α protein and mRNA, TGF-b1)	ELISA and qRT-PCR	5, 10, 20, and 40 mM	24 h	Equivalent anti-inflammatory efficacy of NAC and CH in reducing TNF-α
Corazz et al. 2021 [32] (Brazil)	Randomized clinical trial	NAC (medicament)	G1: CH + saline G2: CH + 2% CHX	36 Teeth with endodontic infection (32 anterior & 4 premolars)	Immunoresolvents, (Resolvins E1, D1)	ELISA	NA	14 days	NAC significantly increased RvE1 and RvD2 in apical periodontitis after 14 days of treatment in comparison to CH
Silveira 2013 [42] (Spain)	In-vitro	NAC 100 mg mL combination with alexidine (2%–0.007 8%)	Alexidine alone	Isolated from a failed endodontic treatment	Microbail *E. faecalis*	CFU	NA	1–5 min	All two groups showed similar result with not statistically significant
Khosravi 2018 [43] (Iran)	In-vitro	G1: Ciprofioxacin and NAC G2: levofloxacin and NAC	G1: normal saline G2: CH G3: ciprofloxacin G4: levofloxacin	120 Humans extracted teeth with single canals	Microbial *E. faecalis*	RCC SEM	NA	1 Week	All intracanal medicaments were significantly more effective than calcium hydroxide (*P* < 0.05). The combination of levofloxacin and NAC caused significantly higher reduction in colony count in comparison with other tested medicaments (*P* = 0.001)

*NAC* N-acetylcysteine, *CHX* Chlorhexidine, *CH* Calcium Hydroxide, *DS* Diclofenac Sodium, *PDT* Photo Dynamic Therapy, CFU Colony forming units, *CLSM* Confocal laser scanning microscopy, *SEM* Scan electron microscope, *NaOCl* Sodium hypochorite, *TNF-a* tumor necrosis factor-alpha, *TGF-b1* transforming growth factor-beta1, *LPS* lipopolysaccharide-induced, *RvE1* Resolvins E1, *RvD2* Resolvins D2, *ELISA* enzyme-linked immunosorbent assay, *ATP* Adenosine triphosphate, *RCC* Reduction in the colony counts, *MTT* 3-(4,5-dimethyl-2-thiazolyl)-2,5-diphenyltetrazolium bromide

### Outcome measures

Thirteen studies [14, 30, 31, 34–43] assessed the antibacterial efficacy of NAC, and two studies [32, 33] assessed the anti-inflammatory efficacy of NAC.

With regards to the target bacteria assessed, *E. faecalis* was evaluated in 13 studies [14, 30, 31, 34–41], *Streptococcus mutants* was evaluated in four studies [14, 31, 37, 38], and *A. naeslundii* and *L. salivarius* were also evaluated by two studies [14, 37]. In most of the included studies, the antibacterial efficacy of NAC was determined by quantifying the viable bacteria (colony-forming units) and the proportion of the dead cells.

### Intervention and comparison groups

In 13 studies [14, 30–41], NAC was the only intervention, while in two studies [42, 43]. NAC was combined with other antibacterial agents. NAC was administered either as irrigation or medicament. Comparison groups varied greatly across the included studies, with most of the studies including more than one comparison group. The most used comparison groups were CHX, calcium hydroxide, and saline (Table 1).

### Main outcomes

#### Antibacterial efficacy

Thirteen studies reported a good antibacterial and antibiofilm efficacy of NAC. Out of these 13 studies, seven studies [14, 30, 31, 36–39] reported better antibacterial efficacy of NAS compared to control groups; two studies [34, 40] reported equivalent antibacterial efficacy of NAC and control groups (CHX in one study and calcium hydroxide in the other); while two studies [35, 41] reported inferior efficacy of NAC compared to the control groups. One study [43] showed that a combination of NAC with Levofloxacin provided greater antibacterial efficacy when compared to Levofloxacin alone, while one study failed to report any added antibacterial effect of ANC when combined with alexidine [42] (Table 1).

#### Anti-inflammatory efficacy

As detailed in Table 1, two studies [32, 33] reported the anti-inflammatory efficacy of NAC. The first study by Corazz et al. assessed the efficacy of NAC and calcium hydroxide on the levels of resolvins (immunosorbent, namely E1 and D2) in apical periodontitis. The results revealed superior efficacy of NAC in increasing the immonosolvents as compared to calcium hydroxide [32]. The other study by Karapinar et al. assessed the anti-inflammatory efficacy of NAC on lipopolysaccharide-stimulated human macrophage cell lines and showed strong efficacy of NAC in reducing TNF-α protein levels which was comparable to calcium hydroxide at the 4th hour. The authors concluded that NAC can be used as an alternative to calcium hydroxide [33].

## Discussion

Several bacterial species have been identified in the oral cavity, and more specifically in association with endodontic infections. Owing to this complexity of the endodontic microbiome, efforts are required to identify potential medicaments or root canal irrigating solutions. In an endeavor to find evidence on the same, we conducted this systematic scoping review of the literature.

Indeed, seeking for an ideal intracanal medicament with antibacterial and anti-inflammatory properties continues in the context of endodontics. This mission is pivotal for the success of endodontic treatment. The ideal intracanal medicament should possess good antimicrobial and anti-inflammatory activities, favorable physical properties, be biocompatible, and be capable of promoting endogenous production of lipid mediators that actively drive the resolution of inflammation.

The antibacterial and antioxidant properties of NAC have received considerable attention recently [44]. The current scoping review revealed that NAC is superior to or, at least, as efficacious as the currently used intracanal medicaments. In the context of the antibacterial/antibiofilm activity, seven out of 11 included studies reported a better antibacterial efficacy of NAC compared to NaOCl and 2% calcium hydroxide. While NAC was equivalent to CHX and calcium hydroxide in two studies. Contrastingly, two studies [35, 41] reported inferior efficacy for NAC compared to taurolidine and calcium hydroxide. It seems that there are minor discrepancies among the results of the included studies which can be attributed to the different methodologies such as concentration of the tested agents, targeted microorganism, and assessment methods.

Anti-inflammatory activity of NAC was assessed in two studies only, and both reported good anti-inflammatory efficacy of NAC versus calcium hydroxide, the gold standard anti-inflammatory intracanal medicament. The anti-inflammatory effect of calcium hydroxide is related to its high pH [34, 40]. However, Corazza et al. [32] reported that calcium hydroxide intracanal medication was unable to increase the levels of resolvins in apical periodontitis, while NAC intracanal medication significantly increased their levels after 14 days of treatment.

The therapeutic action of NAC is ascribed to its thiol group—the active moiety that plays a very important role in scavenging the free radical as well as the destruction of disulfide bonds of bacterial protein ultimately leading to irreversible damage of bacterial growth [27]. For instance, NAC was found to reduce the formation of biofilms by non-oral pathogens such as *Pseudomonas aeruginosa* and *Staphylococcus spp* [45] and *Stenotrophomonas maltophilia* and *Burkholderia cepacia* complex [24]. Furthermore, studies demonstrated that NAC inhibited growth and biofilm formation of oral pathogens such as *Streptococcus mutans, Porphyromonas gingivalis, Aggregatibacter actinomycetemcomitans, Enterococcus faecalis, and P. intermedia* [30, 31, 46]. NAC exerts its antibiofilm/antibacterial effects probably through decreasing biofilm formation, inhibiting bacterial adherence, and reducing the production of extracellular polysaccharide matrix. Overall, the exact mechanisms of antibiofilm/antibacterial activities of NAC have not fully been understood, and experts think of a complex and multifactorial activity [47].

In addition to its antimicrobial effect, NAC is considered a strong anti-inflammatory agent per se*.* It exerts reduction effects on many inflammatory mediators (cytokines) through suppression of nuclear factor kappa B (NF-κB) [48, 49]. Anti-inflammatory effects of NAC are highly augmented by its potent antioxidant activities. Biswas and de Faria (2007) concluded that oxidative stress appears before inflammation as a primary abnormality [50]. Hence, the potent antioxidative properties of NAC make it highly potential as an anti-inflammatory agent. NAC exerts its direct antioxidant activity through its free thiol group that reacts with reactive oxygen and nitrogen species like the hydroxyl radical, nitrogen dioxide, carbon trioxide ion, thiyl radical, nitroxyl -the reduced and protonated form of nitric oxide-, radical anion superoxide, hydrogen peroxide, and peroxynitrite [48]. These free radicals are harmful to the cells, and unless scavenged properly and timely they will lead to the production of pro-inflammatory and inflammatory cascades ending unfortunately with irreversible cell damages. Antioxidation may occur through the endogenous antioxidants led by glutathione and/or through augmentation by exogenous antioxidants such as NAC which is converted in the body into glutathione [48],

The current evidence indicated that NAC is a promising agent as intracanal medicament with favorable antimicrobial and anti-inflammatory properties. However, this evidence does not have a sufficient clinical base to support NAC use as a regular root canal irrigating solution and/or intracanal medicament. Most of the included studies were in vitro, the fact that can’t be relied on to decide the biocompatibility. Worthy to note that the availability of sodium hypochlorite and CH and their reasonable prices, along with the vast clinical research on both of them support keeping their positions as traditional and standard root canal irrigating solutions and intracanal medicament. This will continue until further well-designed, large-scaled clinical research on NAC take place. Another major limitation of this study is that the evidence obtained via scoping review is not as strong as that obtained by systematic review and meta-analysis. Scoping review neither synthesizes the findings from individual studies, nor generates the summary findings, and it lacks mandatory critical appraisal (risk of bias assessment). However, Scoping review is still a useful tool as a resource of evidence synthesis approach, to scope a body of literature, and to clarify the concepts of the main subject to identify the knowledge gap [51]. Meta-analysis is recommended for strong evidence and subsequent decision-making for clinical use of NAC as root canal irrigating solution and/or intracanal medicament. However, this mandates conducting sound primary clinical studies first.

## Conclusions

There is accumulating evidence on the anti-microbial and anti-inflammatory efficacy of NAC in context of endodontics. However, further clinical trials with robust methodology and objective and reliable clinical, biological and microbial outcomes are warranted to translate its use for clinical practice on humans.

## Data Availability

All data generated during this study are included in this manuscript.
