# Correction: Antibacterial and anti-inflammatory efficacy of N-acetyl cysteine in endodontic treatment: a scoping review

**DOI:** 10.1186/s12903-022-02458-x

**Published:** 2022-09-22

**Authors:** Saleem Abdulrab, Nawras Mostafa, Sadeq Ali Al‑Maweri, Hisham Abada, Esam Halboub, Hatem A. Alhadainy

**Affiliations:** 1 Um Salal Health Centre, Primary Health Care Corporation, Doha, Qatar; 2 Department of Restorative Dentistry, Faculty of Dentistry, University of Western Cape, Cape Town, South Africa; 3 College of Dental Medicine, QU Health, Qatar University, Doha, Qatar; 4 Department of Endodontics, Faculty of Oral and Dental Medicine, Kafrelsheikh University, Kafr El‑Sheikh, Egypt; 5 Department of Maxillofacial Surgery and Diagnostic Sciences, College of Dentistry, Jazan University, Jazan, Kingdom of Saudi Arabia; 6 Department of Oral Medicine, Oral Pathology and Oral Radiology, Faculty of Dentistry, Sana’a University, Sanaa, Yemen; 7 Department of Endodontics, College of Dentistry, University of Tanta, Tanta, Egypt

## Correction to: BMC Oral Health (2022) 22:398 10.1186/s12903-022-02433-6

In this article, the affiliations of all the authors were inadvertently swapped. The correct affiliations are listed below:

Saleem Abdulrab1,2*, Nawras Mostafa1, Sadeq Ali Al‑Maweri3, Hisham Abada4, Esam Halboub5,6 and Hatem A. Alhadainy7

1 Um Salal Health Centre, Primary Health Care Corporation, Doha, Qatar.

2 Department of Restorative Dentistry, Faculty of Dentistry, University of Western Cape, Cape Town, South Africa.

3 College of Dental Medicine, QU Health, Qatar University, Doha, Qatar.

4 Department of Endodontics, Faculty of Oral and Dental Medicine, Kafrelsheikh University, Kafr El‑Sheikh, Egypt.

5 Department of Maxillofacial Surgery and Diagnostic Sciences, College of Dentistry, Jazan University, Jazan, Kingdom of Saudi Arabia.

6 Department of Oral Medicine, Oral Pathology and Oral Radiology, Faculty of Dentistry, Sana’a University, Sanaa, Yemen.

7 Department of Endodontics, College of Dentistry, University of Tanta, Tanta, Egypt.

The original article has been corrected.

